# Appearance of *Planktothrix rubescens* Bloom with [D-Asp^3^, Mdha^7^]MC–RR in Gravel Pit Pond of a Shallow Lake-Dominated Area

**DOI:** 10.3390/toxins5122434

**Published:** 2013-12-12

**Authors:** Gábor Vasas, Oszkár Farkas, Gábor Borics, Tamás Felföldi, Gábor Sramkó, Gyula Batta, István Bácsi, Sándor Gonda

**Affiliations:** 1 Department of Botany, University of Debrecen, Egyetem tér 1, Debrecen H-4032, Hungary; E-Mails: Foszi@freemail.hu (O.F.); sramko.gabor@science.unideb.hu (G.S.); gondasandor@gmail.com (S.G.); 2 MTA Centre for Ecological Research, Department of Tisza Research, 18/c. Bem square, Debrecen H-4026, Hungary; E-Mail: borics.gabor@okologia.mta.hu; 3 Department of Microbiology, Eötvös Loránd University, Pázmány Péter sétány 1/C, Budapest H-1117, Hungary; E-Mail: tamas.felfoldi@gmail.com; 4 MTA-ELTE-MTM Ecology Research Group, Pázmány Péter sétány 1/C., H1117 Budapest, Hungary; 5 Department of Organic chemistry, University of Debrecen, Egyetem tér 1., Debrecen H-4032, Hungary; E-Mail: batta@unideb.hu; 6 Department of Hydrobiology, University of Debrecen, Egyetem tér 1, Debrecen H-4032, Hungary; E-Mail: istvan.bacsi@gmail.com

**Keywords:** *Planktothrix*, waterbloom, microcystins, MALDI-TOF, cyanobacteria

## Abstract

Blooms of toxic cyanobacteria are well-known phenomena in many regions of the world. Microcystin (MC), the most frequent cyanobacterial toxin, is produced by entirely different cyanobacteria, including unicellular, multicellular filamentous, heterocytic, and non-heterocytic bloom-forming species. *Planktothrix* is one of the most important MC-producing genera in temperate lakes. The reddish color of cyanobacterial blooms viewed in a gravel pit pond with the appearance of a dense 3 cm thick layer (biovolume: 28.4 mm^3^ L^−1^) was an unexpected observation in the shallow lake-dominated alluvial region of the Carpathian Basin. [d-Asp^3^, Mdha^7^]MC–RR was identified from the blooms sample by MALDI-TOF and NMR. Concentrations of [d-Asp^3^, Mdha^7^]MC–RR were measured by capillary electrophoresis to compare the microcystin content of the field samples and the isolated, laboratory-maintained *P. rubescens* strain. In analyzing the MC gene cluster of the isolated *P. rubescens* strain, a deletion in the spacer region between *mcy*E and *mcy*G and an insertion were located in the spacer region between *mcy*T and *mcy*D. The insertion elements were sequenced and partly identified. Although some invasive tropical cyanobacterial species have been given a great deal of attention in many recent studies, our results draw attention to the spread of the alpine organism *P. rubescens* as a MC-producing, bloom-forming species.

## 1. Introduction

Blooms of photoautotrophic organisms, like algae and cyanobacteria, are well-known phenomena that have been found in many types of fresh and marine waters over the past few decades [1,2]. Near to the spectacular discoloration of the habitats, several unpleasant accompanying incidences were detected with health and economic consequences, such as human and animal poisonings, fish-kills, and decline in quality of drinking water [3]. Many cyanobacterial and algal strains can produce several toxic metabolites with diverse chemistry and bioactivity which may cause these problems [4,5].

While the harmful algal blooms (HAB) are mainly dominated by eukaryotic algal species (*Dinophyceae, Bacillariophyceae*) in marine waters, cyanobacteria occur much more frequently in freshwaters and cause these phenomena [6,7].

Microcystin (MC) as the most frequent cyanobacterial toxin is produced by entirely different cyanobacteria, including unicellular, multicellular filamentous, heterocytic, and non-heterocytic bloom-forming species. MCs are synthesized via non-ribosomal peptide synthetases (NRPS) and polyketide synthases (PKS) assembled into large multifunctional proteins encoded by the *mcy* gene cluster [8]. The general chemical structure of MC is cyclo (d-Ala^1^,X^2^,d-MeAsp^3^,Z^4^,Adda^5^, d-Glu^6^,Mdha^7^), where d-MeAsp is the non-proteinogenic amino acid d-erythro-iso-aspartic acid (methyl aspartate), Mdha is *N*-methyl-dehydroalanine and Adda is an amino acid with a C10-chain: (2*S*,3*S*,8*S*,9*S*)-3-amino-9-methoxy-2,6,8-trimethyl-10-phenyldeca-4,6-dienoic acid. X and Z represent variable L-amino acids in positions 2 and 4, respectively [5].

Recently, progress has been made in the elucidation of the genetic basis of MC synthesis for all three main MC producers occurring in freshwater, *i.e.*, *Anabaena*, *Microcystis* and *Planktothrix*. Three gene clusters responsible for the biosynthesis of MCs, containing 9 or 10 genes (depending on the genus) and spanning 55 kb, have been sequenced. The corresponding genes of *Microcystis aeruginosa* K-139 and PCC 7806, *Planktothrix agardhii* CYA 126, and *Anabaena* sp. strain 90 have been completely sequenced [9,10,11].

*Planktothrix* is one of the most important MC-producing genera in temperate lakes [12]. Of the MC-producing genotypes within this genus, the red-pigmented phycoerythrin (PE)-rich genotypes are assigned to *Planktothrix rubescens,* while the green-pigmented phycocyanin (PC)-rich genotypes are frequently assigned to *Planktothrix agardhii* [13]. Generally, *Planktothrix rubescens* is found in deep, stratified and oligo- to mesotrophic waters in which metalimnetic layers can be built up. *Planktothrix agardhii* has a broader distribution and inhabit shallow, polymictic water bodies in the mesotrophic to hypertrophic nutrient range [1].

*P. rubescens* was reported in the following European subalpine lakes: Zurich (Switzerland), Garda (Italy), Mondsee (Austria), Nantua (France) and Bourget (France) [14,15,16,17,18]. Various chemical, physical, and biological parameters are known to contribute to the developmental and spatial distribution of cyanobacterial populations [1], but the determinism of cyanobacterial blooms and their impact at the lake scale are not clearly understood. 

*Planktothrix* spp. differ in their cellular MC contents as well as the production of MC variants [12,19]. Different MC structural variants were characterized for *Planktothrix* strains isolated from lakes in the Alps: the methyl-dehydro-alanine residue (Mdha) genotype, which was found to synthesize structural variants containing only Mdha in position 7; the butyric acid (Dhb) genotype, which was found to contain Dhb instead of Mdha in the same position; and the homotyrosine (Hty) genotype, which was found to contain Hty and Leu in position 2 but never Arg. The Hty variant has always been found to co-occur with Dhb in position 7 of the molecule [20,21].

Numerous papers have already investigated the impact of various biotic and abiotic environmental factors on MC production by various cyanobacterial strains. These studies demonstrated that MC production can be influenced by temperature, light, nutrients such as nitrogen and phosphorus, pH, iron, xenobiotics, and predators [7,22]. Despite inconsistent results, the production of MCs by the cells seems to be linked to their growth rate, which is itself affected by environmental conditions. On the other hand, several studies on variations in the proportions of MC-producing cells demonstrated the potential influence of nutrient concentrations, light and temperature, suggesting that there is a negative correlation between the proportions of MC-producing cells and the abundance of cyanobacterial cells [23]. 

During the last decade, genetic methods have significantly contributed to our understanding of the distribution of genes that are involved in the production of MCs in cyanobacteria causing cyanobacterial HABs.

The occurrence of inactive *mcy* genotypes (i.e. genotypes possessing the *mcy* genes but lacking MC production) of *Planktothrix* spp. and *Microcystis* spp*.* in nature might be understood as support for the *mcy* gene loss hypothesis. Moreover, inactivation of the *mcy* gene cluster by transposable elements or point mutations might be seen as an intermediate step in reorganization of the *mcy* gene cluster towards cell types with modified MC synthesis [24,25]. 

In this study we report the presence of *P. rubescens* bloom in a wind-sheltered, stably stratified shallow lake. Based on the unusual finding, we claim that *P. rubescens* can occur and build toxic blooms in waters which functionally mimic the deep alpine lakes. The morphometric features of the pond and the relevant physical and chemical variables were studied in order to understand the appearance of this alpine cyanobacterial species in the shallow lake-dominated alluvial region of the Carpathian Basin. In addition to the morphological and molecular identification of the species, we intended to study the toxicity of the species and to analyze the toxin profile by MALDI–TOF and NMR analyses. The *mcy* gene cluster of the isolated strain of the unusual bloom causing *P. rubescens* was also investigated and compared to the sequenced *mcy* gene cluster of strain CYA126/8.

## 2. Results

### 2.1. Physicochemical Parameters of the Study Site

**Figure 1:**
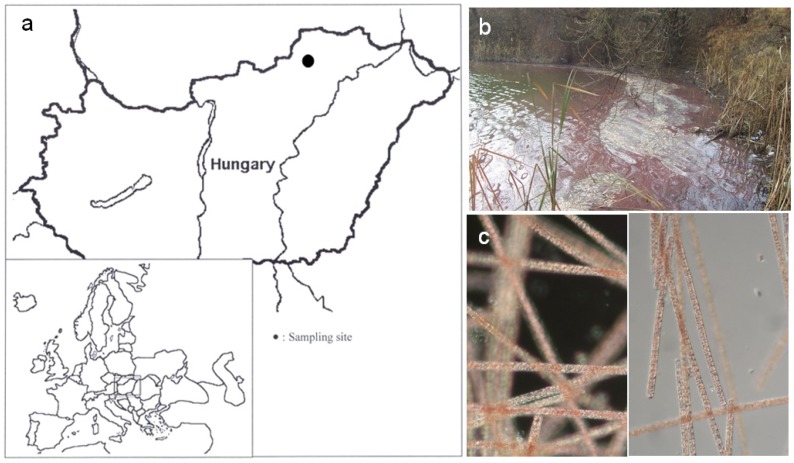
(**a**) Location of Kocka pond in Hungary, indicated by a filled circle; (**b**) The *Planktothrix rubescens* bloom in the gravel pit pond; and (**c**) a microscopic observation of *Planktothrix rubescens* trichomes from the pond.

Analyses of water samples revealed high conductivity and alkaline character of the pond where the water bloom occurred (Figure 1). Physicochemical parameters in the pond during algal blooms are summarized in Table 1. Due to the pond’s small size and leeward location, this type of standing waters are stratified in the vegetation period with a 3 m metalimnion depth [26]. Concentration of nutrients (Table 1) refer to meso-eutrophic character and, at this range, nutrient limitation does not develop [27].

**Table 1:** Morphometric features of the lake and the relevant physical and chemical variables.

Variables	value	unit
Lake area	5.2	(Ha)
Mean depth	3.2	(m)
Max. depth	7	(m)
Lake volume	1.6 × 10^5^	(m^3^)
Secchi transparency	1.2	(m)
pH	8.34	
Specific electrical conductivity	820	(µS cm^−1^)
COD_(sMn)_	15.8	(mg L^−1^)
TOC	22.0	(mg L^−1^)
DOC	15.8	(mg L^−1^)
Inorganic Nitrogen (IN)	1953	(µg L^−1^)
Soluble Reactive Phosphorus (SRP)	3	(µg L^−1^)
Total Nitrogen (TN)	3125	(µg L^−1^)
Total Phosphorus (TP)	370	(µg L^−1^)

### 2.2. Morphology-Based Identification of the HAB Causing Organism

Prior to the molecular analyses, the collected bloom samples were investigated by light microscope (Figure 1). Trichomes were straight, solitary without sheath, and pale purple in color. Cells were cylindrical, not constricted at cross-walls, and mostly isodiametric with a diameter of 6–8 (8) µm. Cells after division were considerably shorter (3–4 µm). All the cells had numerous aerotopes and seemed densely granulated. Most of the filaments had widely rounded terminal cells, the wall of the distal end of these cells were not thickened. Occasionally, some filaments attenuated to the ends and had slightly conical terminal cells with thickened outer cell wall. These morphological features are identical with those characteristic of *Planktothrix rubescens* (DeCandolle ex Gomont) [28].

### 2.3. Molecular Phylogenetic Analyses

**Figure 2:**
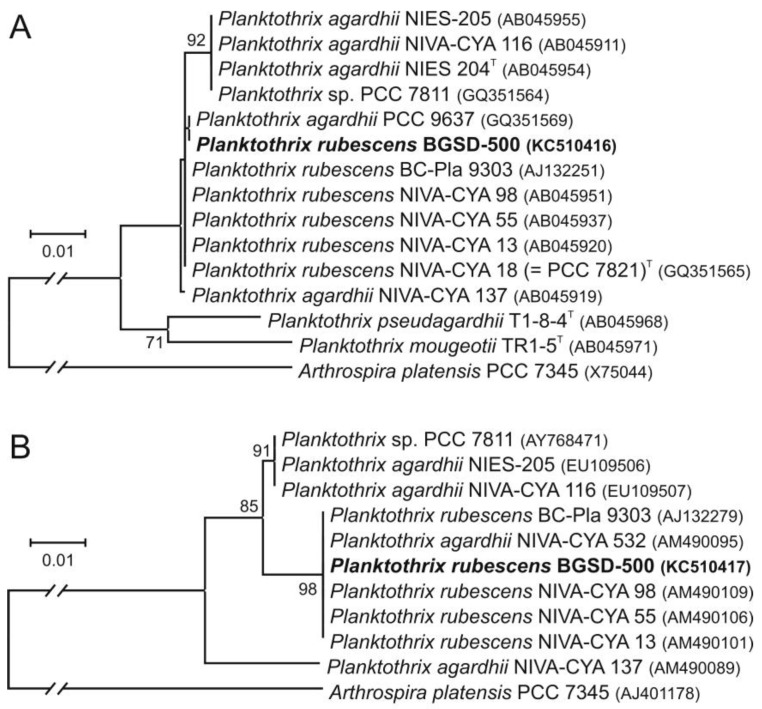
(**A**) Maximum likelihood trees showing the phylogenetic position of BGSD-500 based on the 16S rRNA gene and (**B**) the phycocyanin operon. In the case of the 16S rRNA gene, 1329 nt positions were involved in the analysis that was performed with the HKY + G substitution model, while for the construction of the *cpcBA*-IGS tree, 464 nt were used and the Kimura 2-parameter model was applied. Type strains of *Planktothrix* species according to Suda *et al.* [29] are marked with superscript T. *Arthrospira platensis* PCC 7345 was used as an outgroup in both phylogenetic analyses. Bootstrap values lower than 70 are not shown (based on 500 replicates).

Sequence analysis of regions covering the almost complete 16S rRNA gene and the *cpcBA*-IGS of strain BGSD-500 resulted in 1387 and 527 nt, respectively. Based on the 16S rRNA, BGSD-500 showed high pairwise similarity values (99.9%–100%) to the sequence group containing the type strain *P. rubescens* NIVA-CYA 18 (=PCC 7821)^T^ and was separated from the cluster harboring the type strain of *P. agardhii*, NIES 204^T^ (Figure 2A). The analysis performed with *cpcBA*-IGS sequences showed similar results; BGSD-500 showed 100% pairwise similarity values to the cluster that contained mostly *P. rubescens* isolates (Figure 2B). Unfortunately, no type strain sequences are available currently in databases covering this region, only a shorter fragment with 217 nt from *P. rubescens* NIVA-CYA 18 (=PCC 7821)^T^ (GenBank Acc. No. AJ558154), which was identical with sequences from the aforementioned cluster and showed ≤98.2% pairwise similarity values with the members of the other *cpcBA*-IGS cluster.

### 2.4. Identification of MC and Comparative Analysis of Bloom Sample and the Isolated P. rubescens Strain

**Figure 3:**
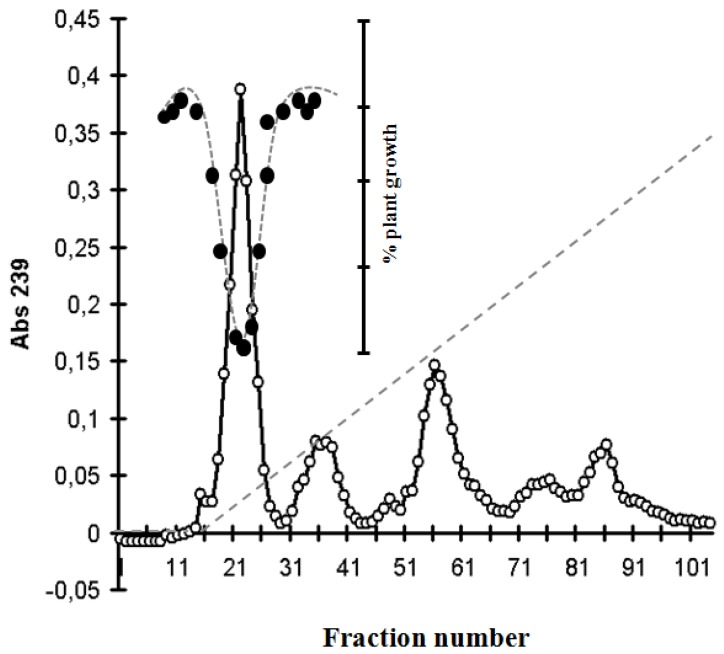
DEAE-52 chromatography and Blue-Green Sinapis Test of [d-Asp^3^, *M*dha^7^]MC–RR from *Planktothrix rubescens*. Absorbance at 239 nm (-○-); hypocotyl length of three-day-old mustard seedlings (-●-), gradient between 0 and 0.2 M NaCl in 5 mM Tris-HCl buffer (---).

Under the purification procedure, the toxic fractions were detected by mustard test (Figure 3). 

**Figure 4:**
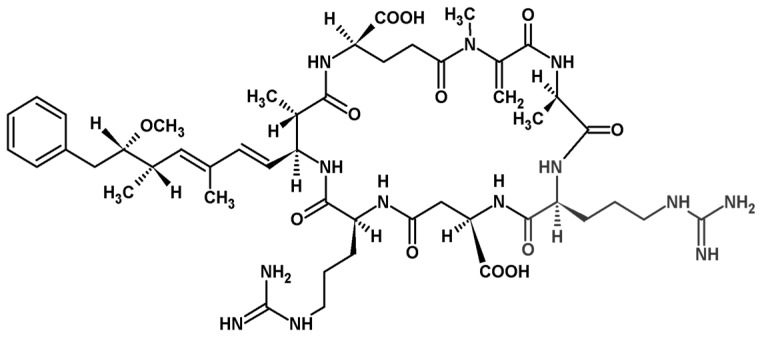
Chemical structure of the identified cyanobacterial heptapeptide [d-Asp^3^, *M*dha^7^]MC–RR.

The main toxic fractions after DEAE cellulose chromatography were combined and further purified by HPLC-DAD. The major toxin was identified as [d-Asp^3^, Mdha^7^]MC–RR (Figure 4) on the basis of the following studies.

The purified MC had an absorption maximum at 239 nm in methanol and exhibited at m/z 1024.6 [MH]^+^ by MALDI-TOF. The constitution of amino acids (Ala^1^,Arg^2^,Asp^3^,Arg^4^,Adda^5^,Glu^6^,Mdha^7^) was confirmed by MALDI post-source decay. Characteristic fragments were: m/z 754 ([Arg^4^-ADDA^5^-Glu^6^-DHB^7^-Ala^1^+H^+^] or [Arg^4^-ADDA^5^-Glu^6^-MDHA^7^-Ala^1^+H^+^]), 714 ([H-Arg^2^-Asp^3^-Arg^4^-ADDA^5^]^+^, lack of Me-Asp^3^), 216 ([Glu^6^-DHB^7^+H^+^] or [Glu^6^-MDHA^7^+H^+^]), 155 ([MDHA^7^-Ala^1^+H^+^] or [DHB^7^-Ala^1^+H^+^]), among others.

The connectivity and configuration of *N*-*methyldehydroalanine* could be determined from TOCSY and NOESY spectra. The Asp^3^ residue showed no methyl group at the C(*β*) position, but rather two H–C(*β*) resonances. This also allowed an assignment of the 1D ^1^H NMR spectrum. 2D HSQC spectra were also recorded. In our sample, an H–C link was identified in the HSQC spectrum between a carbon at 38.0 ppm and 1H at 3.32 ppm, indicating presence of the *N*-methyl group. Also, the =CH_2_ was found, a pair of ^1^Hs at 5.56 ppm and 5.88 ppm located on a ^13^C 116.0 ppm.

Two anabaenopeptin (B, m/z: 837 and F, m/z: 851) congeners were also identified from the *P. rubescens* by MALDI-TOF post-source decay.

**Figure 5:**
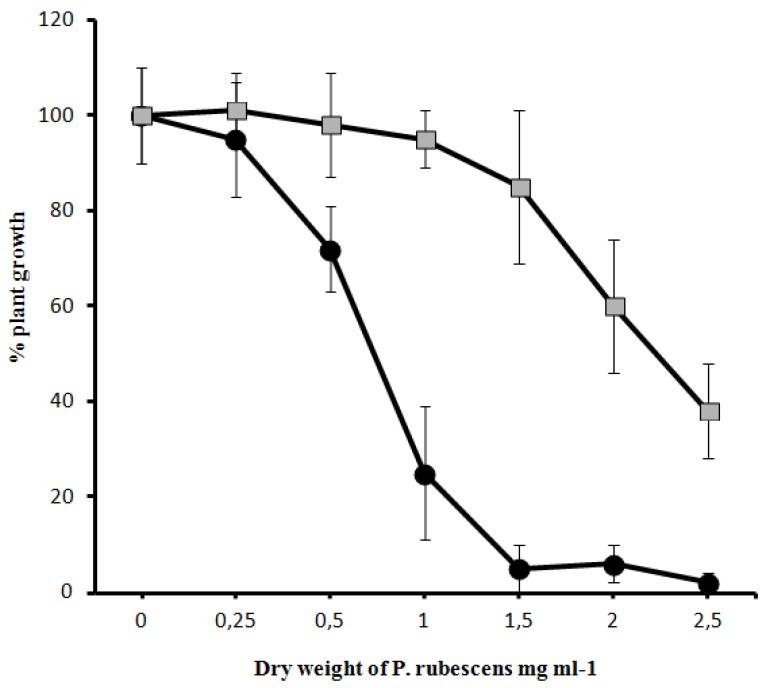
Effect of crude *P. rubescens*-dominated bloom-sample extract (-●-) and the isolated *P. rubescens* BGSD-500 (-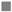-) on the growth of *Sinapis alba* etiolated seedlings (Blue-Green-Sinapis-Test).

The lyophilized samples were tested by mustard test and the toxicity of the samples was calculated. The IC_50_ value of the bloom sample was 0.97, and the BGSD-500 strain was 2.47 (Figure 5). 

**Figure 6:**
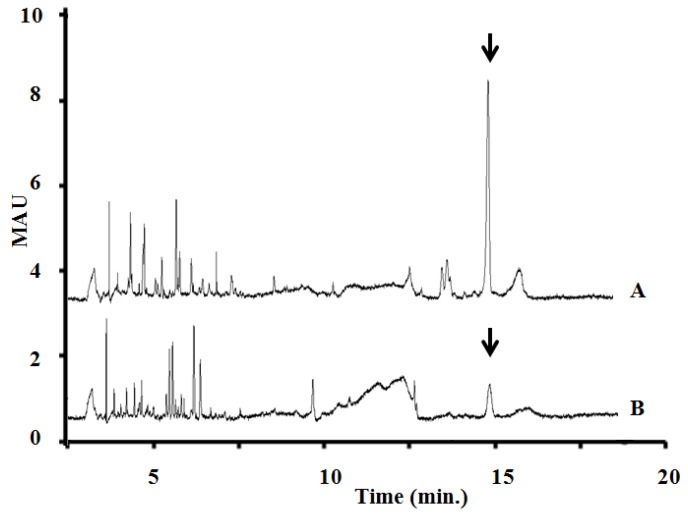
Capillary electrophoresis of *P. rubescens*-dominated bloom-sample extract (A) and the isolated *P. rubescens* BGSD-500 (B). Peak of [d-Asp^3^, *M*dha^7^]MC–RR is indicated by black arrow. (separation conditions: capillary: 64.5 cm, 50 µm i.d., buffer electrolyte: 25 mM borate and 75 mM SDS, pH 9.3, applied voltage: +25 kV, detection: UV absorption at 238 nm).

Comparing the MC content of the samples, the concentration of [d-Asp^3^, Mdha^7^]MC–RR were measured by capillary electrophoresis. The amount of MC content calculated for the bloom sample was 8.57 mg g^−1^, and 1.85 mg g^−1^ for the isolated *P. rubescens* strain (Figure 6).

### 2.5. Analysis of the mcy Gene Cluster

Deletions were identified by shorter-than-expected PCR amplicons at one site. In one case, PCR amplification constantly failed to give amplicons with the corresponding primer pairs (position: 23,612–24,003 nt, [10]. This deletion was located in the spacer region between *mcy*E and *mcy*G, and should therefore not disturb the translation process.

**Figure 7:**
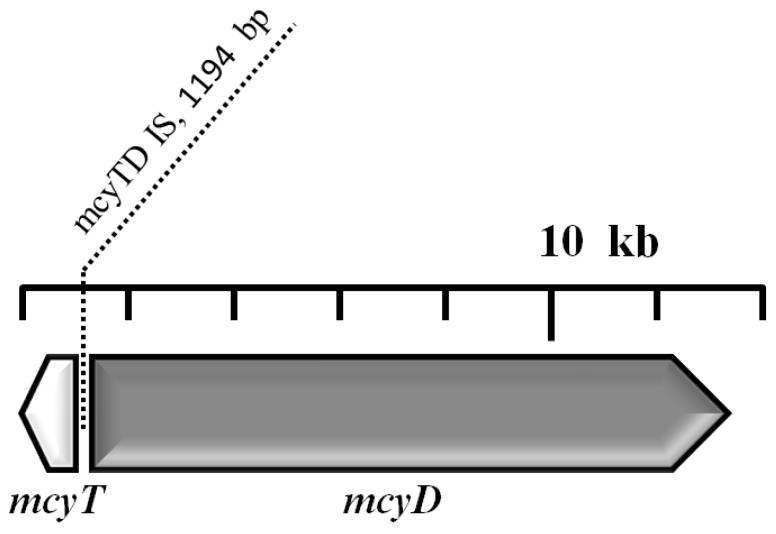
Localization of the detected and partly identified 1194 nt length insertion element in the spacer region between *mcy*T and *mcy*D of *mcy* gene cluster of *Planktothrix*.

The amplification of the MC synthesis gene cluster yielded an unusually long PCR product (around 1.6 kb) when using primer pair myc3 (position: 925–1399 nt); this insertion was located in the spacer region between *mcy*T and *mcy*D. Sequencing of this amplicon yielded 1509 nt and 1387 nt long sequences of the forward and reverse read, respectively, which made it possible to assemble a 1606 nt long “counting” sequence of the region. A standard nucleotide BLAST search in the nucleotide collection of GenBank conducted on 22 June 2013 for highly similar sequences (“megablast”) showed 99% and 98% sequence identity on 17% and 25% of the query length with the MC synthetase-associated thioesterase (*mcy*T) gene of *Planktothrix rubescens* and *P. agardhii*, respectively. When compared to the reference sequence of *Planktothrix agardhii* MC synthesis gene cluster (GenBank accession nr. AJ441056; [10]), the query sequence showed 98% identity on 334 nt length from the 960th to the 1293rd position, then after a *ca*. 1.2 kb gap of an unalignable part, followed by another 98% identical part on 80 nt length from the 1299th to the 1378th position with the same *mcy*T gene. The unalignable region was found to be an insertion into this gene of 1194 nt length (Figure 7).

When searching for highly similar sequences in BLAST (“megablast”), no significant similarity was found for this insertion. Therefore, we repeated the BLAST search but for somewhat similar sequences (“blastn”). This second search has found two somewhat similar sequences in GenBank: the first one was a hypothetical protein of a *Synechococcus* sp. (strain PCC 7002; GenBank accession nr. CP000951) which showed 77% identity on 87% length of the insertion region; whereas the second showed 74% similarity on 75% length in two parts: the first part was similar to signal transduction histidine kinase, while the second part was to tRNA(Ile)-lysidine synthetase of a *Synechococcus* sp. (strain PCC 6312; GenBank accession nr. CP003558). No further similarity was found for the inserted element.

When we compared the sequence of the insert to the whole genome of *Synechococcus* sp. (strain PCC 7002; GenBank accession nr. CP000951) using the LAGAN algorithm [30] in the web-based version of mVISTA [31], it identified a similar part between positions 865,594 and 867,245 of the reference genome, which is potentially homologous to the insert. This region contains 65 nt at the 3'-end of the *icd* gene for the product isocitrate dehydrogenase, NADP-dependent; two hypothetical proteins (corresponding to locus tags SYNPCC7002_A0839 and SYNPCC7002_A0840) in the whole length; and 44 nt at the 5'-end of the *pet*D gene for the product cytb6/f complex subunit IV.

## 3. Discussion

The reddish color of cyanobacterial blooms viewed in Figure 1 in the Kocka pond with the appearance of a dense 3 cm thick layer (biovolume: 28.4 mm^3^/L) was an unexpected observation in our region. The identification of *Planktothrix rubescens* as the dominant bloom-forming species was a surprising observation, because *Planktothrix rubescens* has previously not been identified in our region. 

This species is characteristic in deep-lakes located in Central and Northern Europe [32], including the lakes Zurich, Garda, Mondsee, Geneva, Nantua, Steinsfjorden and Bourget [16,24,33,34,35]. Occasionally the “Burgundy-blood phenomenon” [32] might also occur.

The appearance of the mass on the surface of *P. rubescens* in November is a common phenomenon because during the mixing period, *P. rubescens* is spread within the entire water column but it is usually more concentrated in the upper part of the euphotic zone. During summer stratification, the metalimnetic position is maintained by performing a relatively slow buoyancy regulation [32,36]. Buoyancy is allowed by the production of gas vesicles which is higher when the photosynthetic activity is low. Vertical migration can be stimulated by both light intensity [36] and nutrients distribution [1].

In our case, *P. rubescens* populations thrived under high nitrogen concentrations (Table 1). This interpretation is supported by the observation that *P. rubescens* mass occurrence primarily arises in lakes where Z_eu_ reaches the more stable metalimnetic zone. These lakes are frequently characterized by low phosphate and high nitrogen loads as observed in many oligo- and mesotrophic pre-alpine lakes [33]. Most of Hungary’s territory belongs to alluvial plains where the characteristic lake types are the small, shallow sandhill lakes and oxbows [37]. Natural deep lakes cannot be found in the region, although the deeper oxbows can be stratified stably in the growing season [26,38] in which characteristic vertical distribution of phytoplankton can frequently occur. Nevertheless, gravel and sand mining on the alluvial fans created several pit lakes with maximum depth of 10–40 m. These lakes are stratified, usually mesotrophic and can be characterized by small vertical light attenuation coefficients [39]. These characteristics favor the development of deep chlorophyll maxima (DCM) in the metalimnia by species having capability of effective buoyancy regulation and chromatic adaptation [40]. In the alpine region, DCM is established primarily by the cyanobacteria *Planktothrix rubescens* [41], but the occurrence of this taxon in a mesotrophic pit pond is unique in this region. 

While stratification in deep lakes is a well-known and well-studied limnological phenomenon [42,43,44], there is currently debate about the development and stability of shallow lakes’ stratification [38,45,46]. This is partly due to the lack of clear definition of shallow and deep lakes. Scheffer [47] from a practical point of view proposed the term shallow lakes for those lakes with a depth less than 3 m. Padisák and Reynolds [46] presented a functional approach to delineate a clear difference between shallow and deep lakes. They emphasized that absolute depth alone is not a sound criteria to define shallow or deep lakes; moreover, the stratification is also not decisive, because various stratification patterns can develop in shallow lakes both in space and time. Shallow lakes are generally considered polymictic [47,48,49] because wind-induced mixing continuously sets back the thermal stratification of the shallow water column [50,51]. However, recent studies suggest that even in shallow lakes, periods with stratification can occasionally be observed [26,52]. Intensive study of shallow lakes’ stratification started only in the last few decades [38,45]. Pithart and Pechar [51] found weak stratification in floodplain pools of Lužnice River. Fonseca and Bicudo [53] described a persistent stratification for a few weeks in a tropical shallow reservoir. Folkard *et al.* [54] demonstrated that in sheltered conditions even a small shallow pond can be stratified.

The most successful bloom-forming organisms in a shallow lake-dominated area such as those found in our country are filamentous cyanobacteria: the shade-tolerant species *Planktothrix agardhii* and the invasive *Cylindrospermopsis raciborskii*, as well as the colonial genus *Microcystis* [2,46]. These cyanobacterial genera usually can build waterblooms usually in the summer period, when water temperature reaches 20–25 °C.

Although *Planktothrix rubescens* is a cold-water stenotherm species, it is largely distributed in middle European [1,36] and southern sub-alpine lakes [55]. Considering our report, it cannot be excluded that the organism could appear in any waters that functionally mimic the alpine deep lakes.

During summer stratification it is usually located within the metalimnion [7,44] where it is able to perform an active photosynthetic activity [56].

Sequence analysis of the 16S rRNA gene and the phycocyanin operon has revealed that the closest relatives of the bloom-forming strain BGSD-500 are *P. rubescens* and *P. agardhii* isolates. These two species could not be separated based on the 16S rRNA gene or cell morphology, but could be distinguished based on phycobilin pigment composition [29,57,58]. The main diagnostic feature for differentiation is the high phycoerythrin content that gives a reddish purple or reddish brown color to *P. rubescens* contrary to the blue-green or yellow-green color of *P. agardhii* or *P. suspensa* trichomes [13,29,59]. On the basis of the results of microscopic and nucleotide sequence analyses, strain BGSD-500 was identified as *P. rubescens*.

The results obtained in the Kocka pond thus confirm that the *Planktothrix* bloom sample contained comparably high amounts of MC [12,60]. The MALDI-TOF and the CE-analyses demonstrated that the *P. rubescens* bloom sample and the isolated strain (BGSD-500) primarily contain one main congener, a demethylated variant of MC-RR. This was consistent with other water blooms of *Planktothrix rubescens* reported in the literature [12,24]. After the purification procedure, the pure major component of the microcystin exhibited molecular ion obtained by MALDI-TOF at m/z 1024.6 [M + H]^+^. It was consistent with three previously isolated microcystins: [d-Asp^3^]MC–RR (or reported as [d-Asp^3^,*M*dha^7^]MC–RR) [61,62,63], [Dha^7^]MC–RR [62,63]) and [d-Asp^3^,(*E*)-Dhb^7^]MC–RR [64,65,66]. The isolation of a sufficient amount of the pure compound enabled extensive NMR spectrometric analyses to differentiate between these derivatives. Analysis of 1D and 2D HSQC NMR spectra provided evidence that the molecule is identical to that described by Meriluoto *et al.*, [61], [d-Asp^3^,*M*dha^7^]MC–RR, isolated from *Oscillatoria (Planktothrix) agardhii*. 

This observation concurs with previous studies, describing demethylated variants of MC-RR to be the predominant MC congeners of *P.* *rubescens* [12,20,63,64], accompanied by a varying number of characterized MC variants, such as [Asp^3^]-MC-LR, [Asp^3^]-MC-HtyR and [Asp^3^]-MC-YR, and as yet uncharacterized congeners [12,20].

Freshwater cyanobacterial toxic blooms are a common problem in many Hungarian ponds and lakes. This phenomenon, which has been widely reported in the literature during the last decades, involves many species, including *Microcystis aeruginosa*, *Cylindrospermopsis raciborskii, Chrysosporum (Aphanizomenon) ovalisporum, Planktothrix agardhii* [67,68,69,70,71,72]. Thus far, the presence of MCs in Hungarian shallow lakes has been primarily attributed to *Microcystis aeruginosa* and occasionally to *Planktothrix agardii*. Although almost every investigated *M. aeruginosa* population was an MC producer so far and contained variable amounts of this heptapeptide, the *P. agardii*-dominated blooms have lower MC concentrations due to the patchy distribution of *mcy* genes in *P. agardhii* populations [73]. The present report is the first unambiguous evidence of MC production by *P. rubescens* in our region and reveals that MC production might be more widespread within the cyanobacterial taxa found in Hungarian freshwaters than was previously assumed.

Under the purification procedure the MC was detected by a *Sinapis* plant test, which was then developed by us for the detection of cyanobacterial toxins [67]. The detected MC congener was less toxic than the earlier investigated MC-LR and YR. The impact of structural differences on acute toxicity has also been observed with mouse bioassay and *T. platyurus* bioassay and these results reflected the specificity of sensitivities for different organisms [65]. The lack of congruence between protein phosphatase (PP) inhibition and toxicity of different congeners of MC indicate that other mechanisms, such as uptake, transport, detoxification, other target sites *etc.*, may have a strong modulating effect on the overall toxicity for an animal and can offset or even reverse the specific PP inhibitory activity [64].

The isolated *P. rubescens* culture was shown to contain MC corresponding to an amount of 1.85 MC-LR equiv. mg^−1^ dry weight using CE-analysis. Comparing the concentration of the MC congener in the bloom sample and the isolated strain it can be clearly seen that the bloom sample contained five times more MC than the isolated strain did. This difference may due to specific environmental conditions or to the fact that natural populations are a mixture of different strains with different toxic potentials.

Sequence analysis of the *mcy* region by Christiansen *et al.* [10] revealed a 55 kb cluster of 9 genes presumably involved in MC biosynthesis in *P. agardhii* CYA 126/8. It showed both remarkable similarity to, but also differences from, the completely sequenced *mcy* gene cluster of *M. aeruginosa*. Eight of these genes (*mcy*A, -B, -C, -D, -E, -G, -H, and -J) showed significant similarity to the *mcy* genes from *M. aeruginosa* encoding peptide synthetases (PKSs) and modifying enzymes. One of the main differences between the *mcy* gene clusters of the two aforementioned genera are the general arrangement and the transcriptional orientation of the *mcy* genes which could be explained by deletion or rearrangement of several genes. This is confirmed by the fact that *mcy*F and *mcy*I are lacking in the *Planktothrix* cluster, while *mcy*T is missing in the *Microcystis* cluster. With the help of PCR products of 28 primer pairs covering the whole *mcy* gene cluster in *Planktothrix* we confirmed the presence of the *mcy* gene cluster in our isolates. There were no striking differences in size in the PCR products on agarose gels compared to the corresponding PCR products obtained from strain CYA126/8, except for two positions.

Deletions were identified by shorter-than-expected PCR amplicons at one site, since PCR amplification constantly failed to give amplicon with primer pairs corresponding to the positions from 23,612 to 24,003 nt within the *mcy* cluster of *P. agardhii* CYA 126/8. This deletion was located in the spacer region between *mcy*E and *mcy*G; therefore, it should not disturb the translation process.

Insertions were detected at one site by significantly larger-than-expected PCR products, using the primer pair amplifying the region between positions 925 and 1399 nt, binding to the spacer region between *mcy*T and *mcy*D.

Several *Planktothrix* strains that were inactive in MC synthesis were investigated, and a few were found to contain mutations within the *mcy* gene cluster by deletion(s) and insertion(s) [24,25]. However, some of the investigated strains without detectable MC did not reveal insertions or deletions, and, consequently, they may have acquired point mutations within the *mcy* gene cluster, as suggested by the authors [74,75]). That study was the first showing that mutations do occur frequently within the *mcy* gene cluster and that a large proportion of mutations are caused by insertion of an IS element at different sites [74,75]. 

Although we detected an insertion element in our isolate, its position was in an intergenic region and not likely to disturb the translation process. The detected IS element was close to the *mcy*T region which is a unique *mcy* region in the genus *Planktothrix*. In *Planktothrix*, the *mcy*T gene is located at the 5'-end of the *mcy* gene cluster, but has not been found in the *mcy* gene cluster of other MC-producing cyanobacteria [10,76]. To demonstrate the role of *mcy*T in MC synthesis, the *mcy*T gene was inactivated by experimental mutagenesis in *P. agardhii* strain CYA126/8. The insertional inactivation of *mcy*T resulted in a reduction of MC synthesis by 94% ± 2% (1 SD) compared with the wild type [10]. In contrast, the proportion of MC variants, cellular growth rates, as well as the transcriptional rates of other *mcy* genes were not altered. According to the data of Mbedi *et al.* [77], *mcy*T and *mcy*TD are inadequate regions for the detection of MC-producing *Planktothrix* in field samples, since they also occur in non-producers.

Recombination has been recognized as a general feature in the formation of *mcy* gene clusters for the synthesis of new structural variants of MC and could be modified the net MC amount [74,75]. For the identification of the IS elements we tried to find similar sequences in GenBank. The first one was a hypothetical protein of a *Synechococcus* sp. The second showed 74% similarity on 75% length in two parts: the first part was similar to signal transduction histidine kinase, while the second part was to tRNA(Ile)-lysidine synthetase of a *Synechococcus* sp.

Although our element has probably no influence on the MC synthesis, considering the function of the product of the partly similar sequences, it is worth discussing this possibility. Lysidine is an essential modification that determines both the codon and amino acid specificities of tRNA(Ile) [78] and the signal transduction histidine kinase play a role in signal transduction across the cellular membrane [79]. Both functions could be associated with a regulation of a metabolite production.

## 4. Experimental Section

### 4.1. Site Description and Sampling

The Kocka pond (Figure 1) is a small, shallow, well-sheltered, gravel pit pond with a maximum depth of 7 m, situated in the northeastern part of Hungary (48°08′38.72″; 20°48′06.63″) 111 m a.s.l.. Concentration of the nutrients (Table 1) refers to meso-eutrophic character and, at this range, nutrient limitation does not develop [27]. The pond is used for angling activity with regular fish stocking. Strong red-colored water-bloom was observed and field samples were collected on 19 November 2006.

Samples were taken from the water surface at the center of the pond, where the filaments were associated into mass and covered the water surface in 1–2 cm thick layer. Five L net samples were collected for the toxin analyses and 0.1 L for the isolation of the water-bloom-causing species. The isolated strain was identified as *Planktothrix rubescens* (see below), coded as BGSD-500 and cultivated in BG11 medium at 22 °C with continuous irradiation (50 µmol m^2^ s^−1^). Strain was harvested by centrifuge at 13,000 rpm for 10 min, and the pellet was lyophilized.

### 4.2. Physical and Chemical Variables

Water temperature was measured at the field using a mercury bulb thermometer. The other variables were determined in the laboratory. Samples were kept at 4 °C in darkness until the start of measurements. A pH meter with a glass electrode (WTW pH 539) was used to measure pH based on MSZ 1484-22:2009 Hungarian Standard. The specific electrical conductivity was determined on basis of MSZ EN 27888:1998 Hungarian Standard, using WTW LF539 conductivity meter. Both variables were temperature corrected (20 °C). Sampling, preservation and the analyses were carried out on basis of the Hungarian Standard, MSZ ISO 5813:1992. This standard is equivalent in technical content and fully corresponds in presentation to the International Standard ISO 5813:1983 and to the European Standard EN 25813:1992. For measuring chemical oxygen demand (COD), potassium permanganate was used as an oxidizing agent (MSZ 448-20:1990 Hungarian Standard). Ammonium concentration was determined by a manual spectrophotometric method based on the MSZ ISO 7150-1:1992, Hungarian Standard. This standard is totally equivalent in technical content and fully corresponds in presentation to the International Standard ISO 7150-1:1984. Determination of nitrate concentration was based on colorimetry by titration of salicylic acid (MSZ 1484-13: 2009). Colorimetry was used also to determine nitrite concentration applying sulfonic acid and aminonaphthalene reagents (MSZ 1484-13:2009). Inorganic nitrogen was calculated as the sum of these three forms. Total organic carbon (TOC) measurements were performed with an Elementar High TOC analyzer according to the combustion-infrared method as described in the MSZ EN 1484:1998 standard. Dissolved organic carbon (DOC) measurements were made on filtered water samples (0.45 nm pore diameter). Inorganic phosphorus concentrations were measured by the acid molybdate method (MSZ 448-20: 1990 Hungarian Standard). 

### 4.3. Identification of HAB Species

The HAB species (Figure 1) was identified by observations of morphological characteristics [28]. Phytoplankton samples (50 mL) were preserved with acidic Lugol’s solution and filaments were counted using a particle counter (HIAC/ROYCO 9064) calibrated by manual counting (of at least 400 cells) using an inverted microscope (LEICA DMIL research microscope equipped with DIC and phase contrast techniques). The identification by external characteristics of the species was also done from preserved and unpreserved samples.

### 4.4. Molecular Phylogenetic Analyses

Total genomic DNA was isolated from lyophilized cells according to the liquid nitrogen cell disruption protocol described by Somogyi *et al.* [80]. PCR amplification of almost full length 16S rRNA gene was conducted as given in Lamprinou *et al.* [81], while a region within the phycocyanin operon (*cpcBA*-IGS) was amplified as described in Felföldi *et al.* [82]. Sequencing reactions and capillary electrophoresis were performed by the Biomi Ltd. (Gödöllő, Hungary). The manual correction of automatic base calling on chromatograms and the removal of primer sequences were conducted with the Chromas software v1.45 (Technelysium, Brisbane, QLD, Australia). Sequence alignment (containing sequences obtained from the GenBank database) was performed with SINA [83] in the case of the 16S rRNA gene, and with the built-in Clustal W module in the MEGA5 software [84] in the case of *cpcBA*-IGS sequences. Phylogenetic analyses (including the search for the best-fit models) were performed with MEGA5. Obtained sequences are available under accession numbers KC510416 (16S rRNA gene) and KC510417 (*cpcBA*-IGS).

### 4.5. Purification of MC from Field Samples

Filaments of *P. rubescens* harvested by centrifugation at laboratory temperature (10,000 g, 10 min, Beckman Avanti J-25) were used for MC isolation. Frozen cell pellet (25–30 g) was thawed and frozen again and this procedure was repeated twice. The final thawed cell suspension was lyophilized and extracted overnight with 80% methanol (1:3 mass–volume) at 4 °C with continuous stirring. After centrifugation the pellet was washed twice with 100 mL 80% methanol. The combined supernatants were concentrated in a rotary evaporator at 40 °C (Büchi Rotavapor-R). 

The residue was dissolved in 5 mM Tris-HCl, pH 7.5 and after centrifugation the solution was loaded onto a DEAE column (3 × 15 cm, DE-52, Whatman) equilibrated with 5 mM Tris-HCl, pH 7.5. The column was washed with this buffer and eluted with a gradient between 0 and 0.2 M NaCl in 5 mM Tris-HCl buffer, pH 7.5. The absorbance of the fractions (5–6 mL) was measured at 239 nm in a Shimadzu 1601A spectrophotometer. The plant growth inhibitory effect of fractions was measured with the *Sinapis* test on microtiter plates. Aliquots (20 µL) of the fractions were evaporated to dryness in the wells of microtiter plates and into those wells, an amount of 100 µL of agar containing (1%) plant growth medium was pipetted. 

Based on the mustard growth inhibition, the cyanotoxin-containing fractions were combined and lyophilized, dissolved in methanol, and loaded to a semipreparative C-18 HPLC column (Supelcosil TM SPLC-18, 25 cm, 310 mm, 5µm), and the separation of the compounds was followed at 239 nm by their characteristic absorption spectra, using the gradient according to Chorus and Bartam [7].

The distinctive peaks (with characteristic UV spectra) of the chromatogram was tested with the S*inapis* test and collected for further analysis. Bloom sample and the isolated *P. rubescens* strain were also tested with *Sinapis* test [67].

### 4.6. Identification of MC Congener

#### 4.6.1. MALDI-TOF MS Analysis

Although HPLC-MS analysis is the most common and sensitive method in microcystin measurements, the MALDI-TOF analysis is also a well-known method in the identification of MC congeners [85].

The purified MC was examined in positive-ion mode using a Bruker Biflex MALDI-TOF mass spectrometer equipped with delayed-ion extraction. A 337 nm nitrogen laser was used for desorption/ionization of the sample molecules. Spectra from multiple (at least 100) laser shots were summarized using 19 kV accelerating and 20 kV reflectron voltage. External calibration was applied using the [M + Na]^+^ peaks of malto-oligosaccharides dp 3–7, m/z values 527.15, 689.21, 851.26, 1013.31, and 1175.36, respectively. The measurement was performed in 2,5-dihydroxybenzoic acid (DHB) matrix, by mixing 0.5 mL of matrix solution with 0.5 mL of sample on the sample target and allowing it to dry at room temperature. DHB matrix solution was prepared by dissolving DHB (10 mg) in a mixture (0.5 mL) of ethanol and water (1:1, V:V). The compounds were identified on the basis of the mass of [M + H]^+^ peak. After determination of mass values, post-source decay (PSD) measurements were performed directly from the same sample on the template and MC and other peptides were identified by PSD fragment structure analysis [85].

#### 4.6.2. NMR

NMR spectra were acquired using a Bruker DRX-500 spectrometer operating at 500.13/125.79 MHz for protons and 13 C, respectively. Normal, one-dimensional ^1^H-NMR spectra were obtained in D_2_O at 298 K. Residual HDO signal was saturated. A two-dimensional ^1^H–^13^C HSQC experiment yielded a1 H/^13^C assignment identical to that published by Meriluoto *et al.* ([61], data not shown). 

### 4.7. Capillary Electrophoresis of Field Samples and Isolated P. rubescens Laboratory Strain Samples

Microcystin variants in the whole extracts of the samples were analyzed by micellar electrokinetic chromatography developed by our laboratory [40,41] (separation conditions: capillary: 64.5 cm, 50 µm i.d., buffer electrolyte: 25 mM borate and 75 mM SDS, pH 9.3, applied voltage: +25 kV, detection: UV absorption at 238 nm).

### 4.8. Genetic Analysis of the mcy Gene Cluster

DNA extraction from strains and field samples was performed by a standard phenol-chloroform procedure. PCR amplifications were performed in reaction mixtures of 20 µL as published by Kurmayer *et al.* [20] and Christiansen *et al.* [25]. In order to screen the complete *Planktothrix mcy* gene cluster, 28 primer pairs covering the whole *mcy* gene cluster were used to amplify fragments of 2 kb without interruption [10]. DNA mutations were detected via the difference in PCR product sizes in agarose gels compared to the corresponding PCR products obtained from strain CYA126/8, whose *mcy* gene cluster has been sequenced [10]. The PCR thermal cycling protocol included an initial denaturation step at 94 °C for 3 min, followed by 35 cycles of denaturation temperature of 94 °C for 30 s, annealing temperature of 60 °C for 30 s, and elongation temperature of 72 °C for 2 min. The primer pairs of Christiansen *et al.* [10,25,86], amplifying fragments of *ca*. 500 bp, were used to detect size differences in the cluster. PCR-products with possible inserted or deleted elements were sequenced directly from the same PCR products (sequencing followed the same procedure described in phylogenetic analyses).

## 5. Conclusions

In this multidisciplinary study we reported the presence of *P. rubescens* bloom from a wind-sheltered, stably stratified shallow lake with low phosphate and high nitrogen loads, where the Secchi transparency was 1.2 m. The reddish color of cyanobacterial blooms was an unexpected observation in our region and the causative organism was identified by classic morphological markers and by 16S rRNA gene and phycocyanin operon (*cpcBA*-IGS) as molecular markers. 

The results obtained in the Kocka pond thus confirm that the *Planktothrix* bloom sample contained comparably high amounts of MC. The MALDI-TOF and the CE-analyses demonstrated that the *P. rubescens* bloom sample and the isolated strain (BGSD-500) primarily contain one main MC congener, a demethylated variant of MC-RR. Analysis of MALDI-TOF spectra and 2D HSQC NMR spectra provided evidence that the molecule is identical to [d-Asp^3^,Mdha^7^]MC–RR. 

Comparing the concentration of the MC congener in the bloom sample and in the isolated strain it can be clearly seen that the bloom sample contained five times more MC than the isolated strain. This difference may due to specific environmental conditions but it is important to note a deletion in the spacer region between *mcy*E and *mcy*G, and an insertion were detected at one site binding to the spacer region between *mcy*T and *mcy*D. Although our element has probably no influence on the MC synthesis, considering the function of the product of the partly similar sequences, it is worth discussing this possibility.

Although some invasive tropical cyanobacterial species have recently come to the fore in many studies, in our paper, we draw the attention to the possible spread of an alpine harmful organism, *P. rubescens*.
